# Supplementary material for: Autism gene variants disrupt enteric neuron migration and cause gastrointestinal dysmotility
Source: Nat Commun. 2025 Mar 6;16:2238. doi: 10.1038/s41467-025-57342-3 (PMC11885846; doi:10.1038/s41467-025-57342-3)
Supplement: Supplementary file 1 — Supplementary Information [file 41467_2025_57342_MOESM1_ESM.pdf]

Supplementary Materials for

Autism gene variants disrupt enteric neuron migration and cause gastrointestinal dysmotility

Kate E. McCluskey, Katherine M. Stovell, Karen Law, Elina Kostyanovskaya, James D. Schmidt, Cameron R. T. Exner, Jeanselle Dea, Elise Brimble, Matthew W. State, A. Jeremy Willsey, Helen Rankin Willsey

Corresponding author: helen.willsey@ucsf.edu

The PDF file includes:

Supplementary Figures 1-6

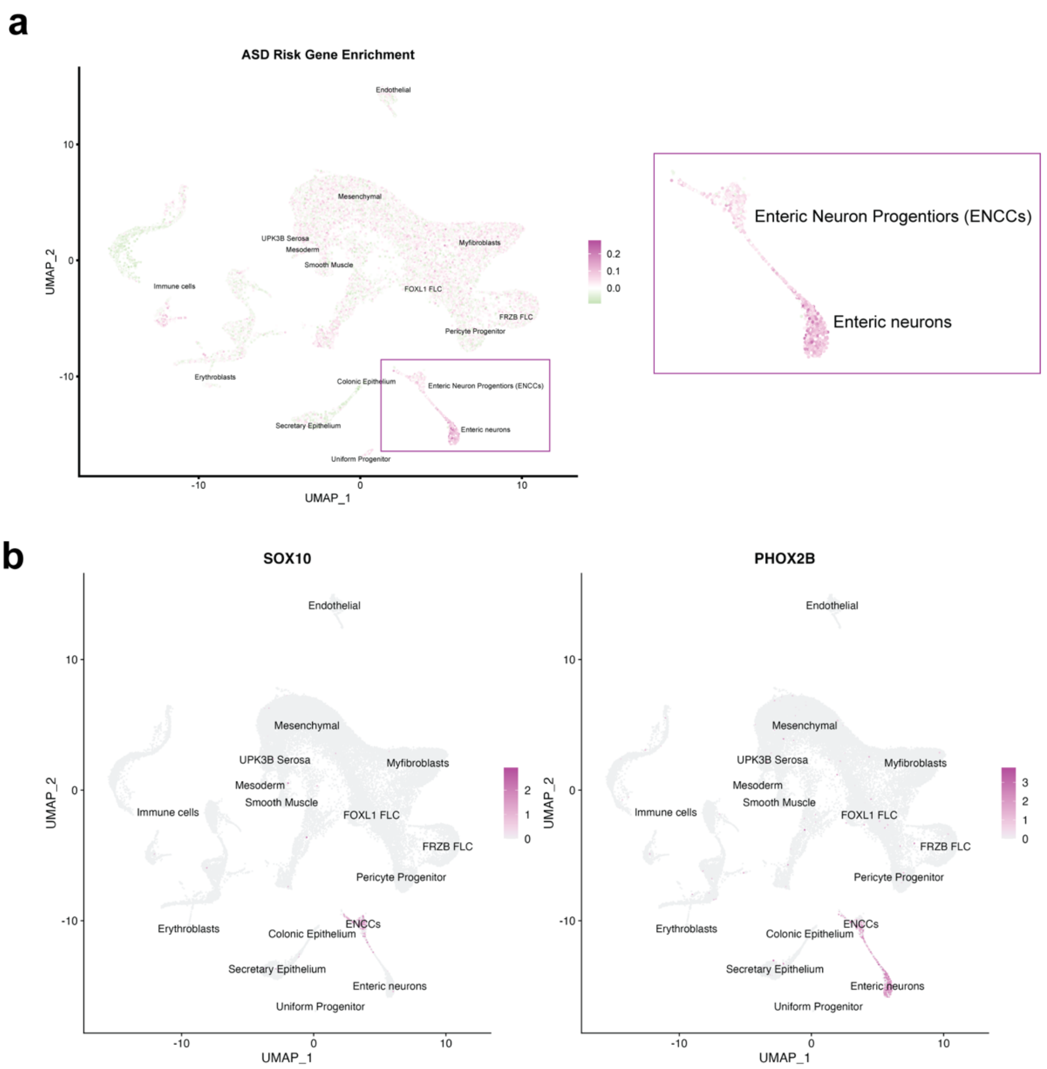

Supplementary Figure 1. hcASD gene expression enrichment in human prenatal gut transcriptomic dataset[26]. Related to Figure 1. (a) UMAP of human prenatal gut scRNA-sequencing data[26]. Cells colored according to enrichment of hcASD gene module scores. Enteric neurons and ENCCs are outlined in a red rectangle and enlarged to the right. (b) The expression patterns of two marker genes *SOX10* (neural crest) and *PHOX2B* (vagal neural crest, ENCCs, and enteric neurons) to show cell-type identities for these populations.

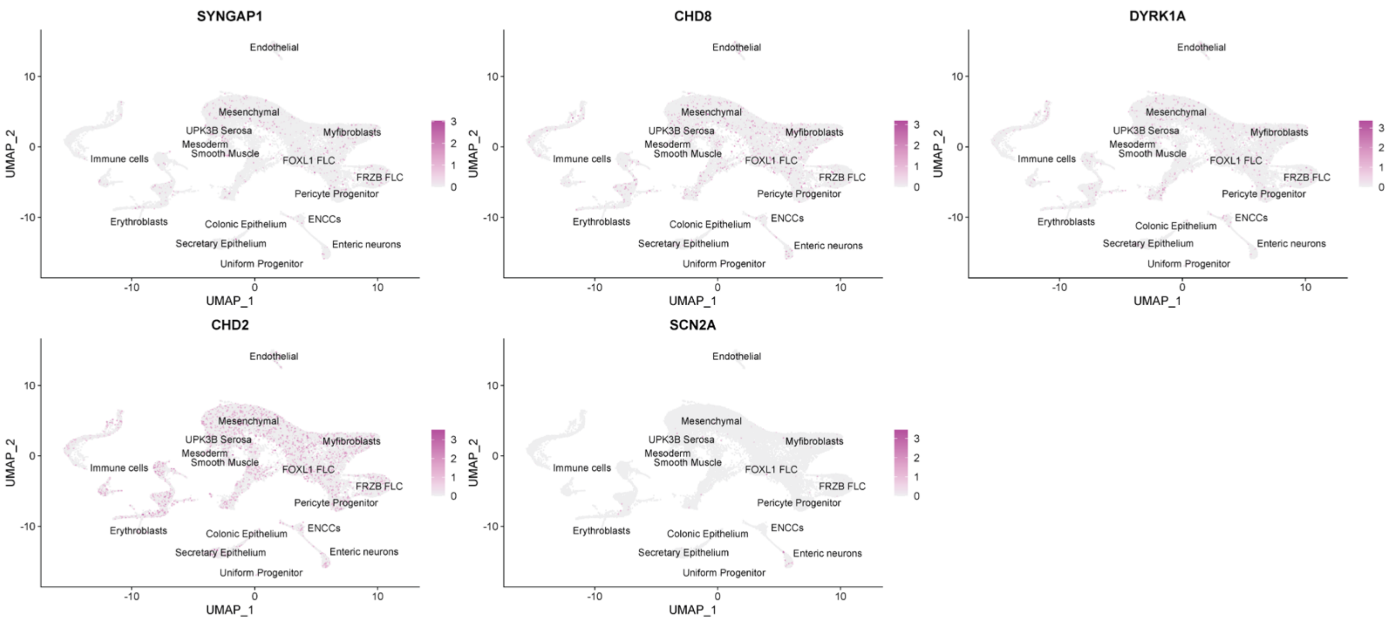

Supplementary Figure 2. hcASD gene expression in human prenatal gut transcriptomic dataset[26]. Related to Figure 1. UMAPs of *SYNGAP1*, *CHD8*, *SCN2A*, *CHD2*, and *DYRK1A* expression.

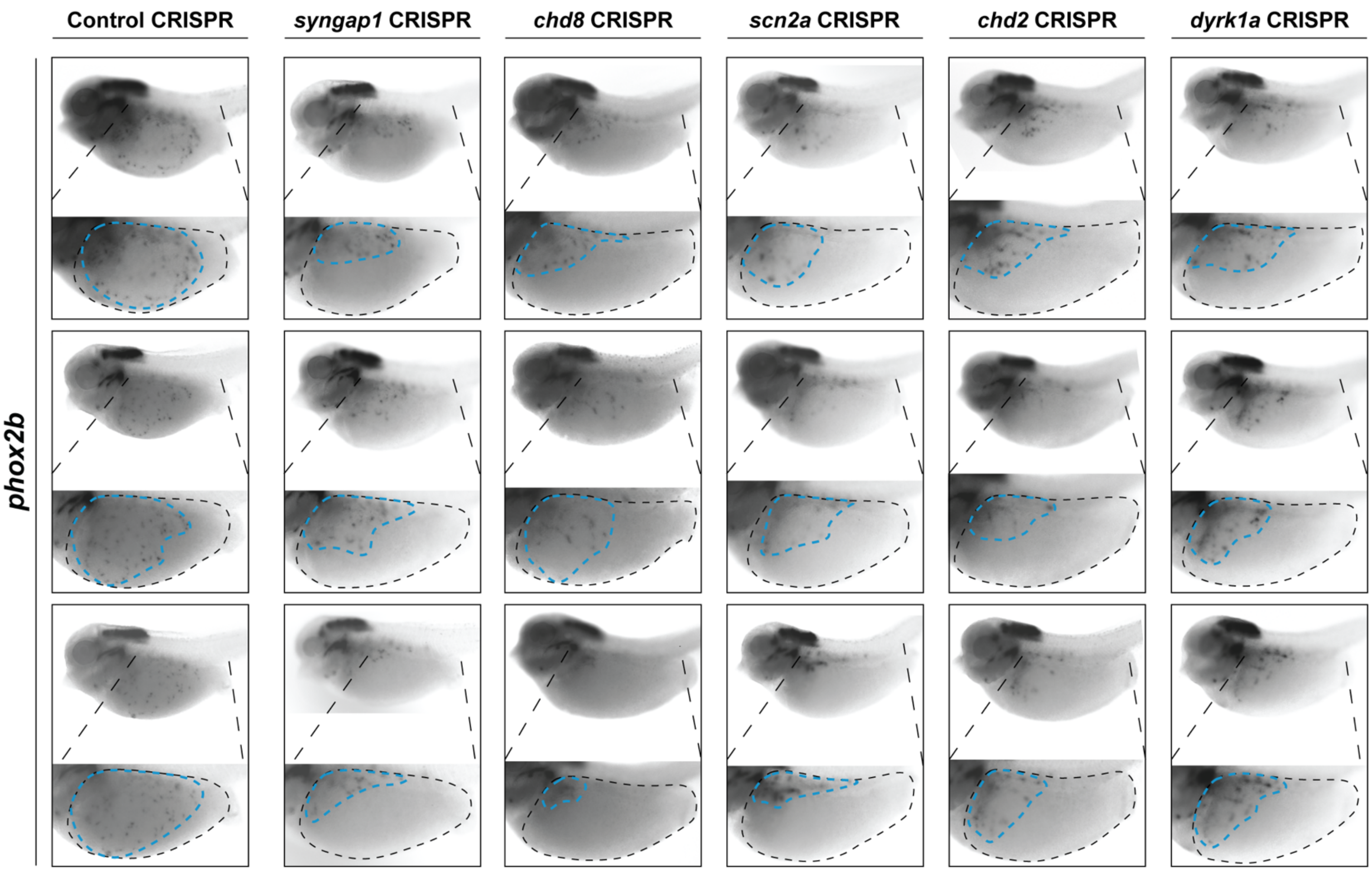

Supplementary Figure 3. Additional representative images of hcASD gene knockdowns. Related to Figure 2. CRISPR mutagenesis of hcASD genes *syngap1, chd8, scn2a, chd2,* or *dyrk1a* show reduced area of ENCC migration but with no consistent migratory patterns between genes.

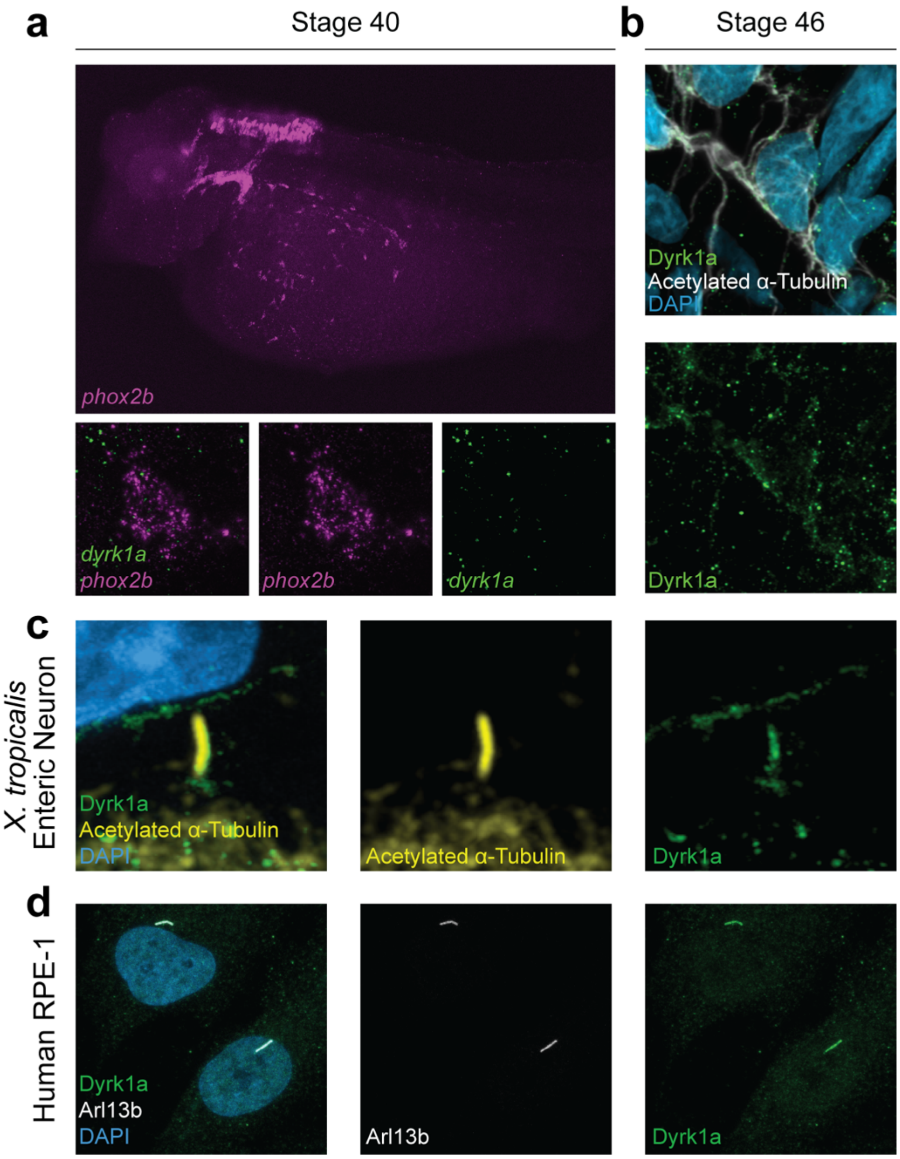

Supplementary Figure 4. *dyrk1a* is expressed in ENCCs and Dyrk1a localizes to microtubule-based structures. Related to Figure 2. (a) RNA *in situ* hybridization chain reaction staining shows *dyrk1a* (green) expression in *phox2b*-positive (magenta) ENCCs in *X. tropicalis*, stage 40. Bottom panel shows a high magnification image of *dyrk1a* mRNA transcripts within a single *phox2b*-positive cell. (b) Immunofluorescence staining shows Dyrk1a (green) localizing to enteric neuron projections in *X. tropicalis* as marked by Acetylated α-Tubulin (white). (c) Immunofluorescence staining shows Dyrk1a (green) localizing to the primary cilium of an enteric neuron in *X. tropicalis* as marked by Acetylated α-Tubulin (yellow). (d) Immunofluorescence staining shows localization of Dyrk1a (green) to the primary cilium of human retinal pigmented epithelial (RPE-1) cells as marked by Arl13b antibody staining (white).

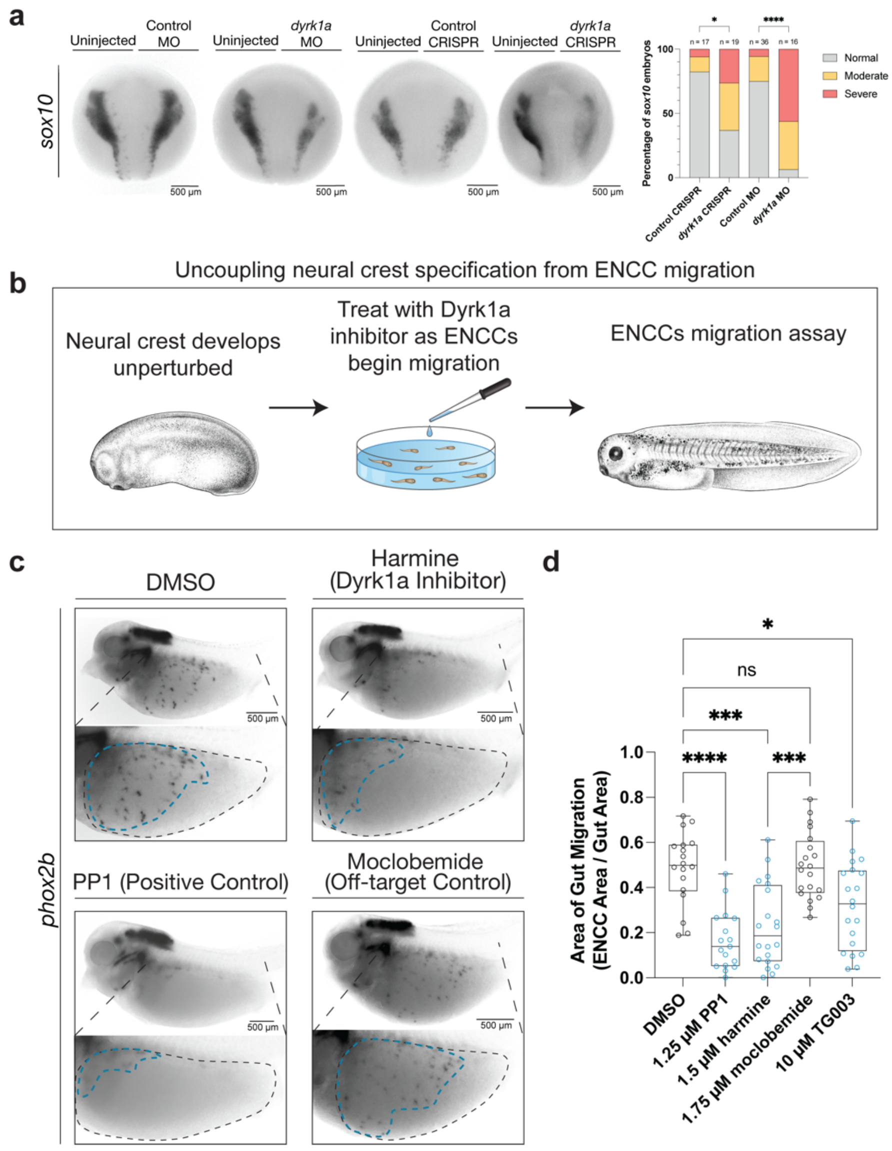

Supplementary Figure 5. *dyrk1a* is independently required for early neural crest development and for later ENCC migration. Related to Figure 3. (a) Perturbation of *dyrk1a* with either CRISPR or morpholino (MO) injection results in an increased proportion of moderate and severe early (stage 15) neural crest phenotypes compared to controls. Migratory neural crest was visualized with RNA *in situ* hybridization staining for *sox10* at NF stage 15. There are significantly more moderate and severe phenotypes in *dyrk1a* CRISPR and MO embryos than controls (Fisher's exact test, CRISPR $p = 0.02$, MO $p < 0.0001$). (b) Schematic of assay to uncouple early neural crest development disruption from later ENCC migration. Tadpoles are raised unperturbed until NF stage 25 when the vagal neural crest begins migration. They are then treated with a Dyrk1a inhibitor or control small molecule and fixed and stained at NF stage 40 to assay ENCC migration. *Xenopus* illustrations © Natalya Zahn (2022)[70]. Drug treatment illustration created by Sarah Pyle. (c) Treatment with Dyrk1a inhibitors harmine or TG003 as well as positive control Ret inhibitor PP1 lead to reduced ENCC migration separately from neural crest specification, compared to DMSO and moclobemide controls. Moclobemide is a monoamine oxidase inhibitor that controls for potential off-target inhibition of monoamine oxidase by harmine. (d) Area of gut migration quantification by condition. Controls in gray, Dyrk1a inhibitors in blue. A Kruskal-Wallis test was performed followed by Dunn's test to adjust for multiple comparisons. p values are shown in which ns (not significant) indicates padj > 0.05. Compared to DMSO (N = 18), PP1 (N = 17, padj < 0.0001), harmine (N = 20, padj < 0.0002), TG003 (N = 20, padj = 0.0435), and compared to moclobemide (N = 20), harmine padj = 0.0001. Source data are provided as a Source Data file.

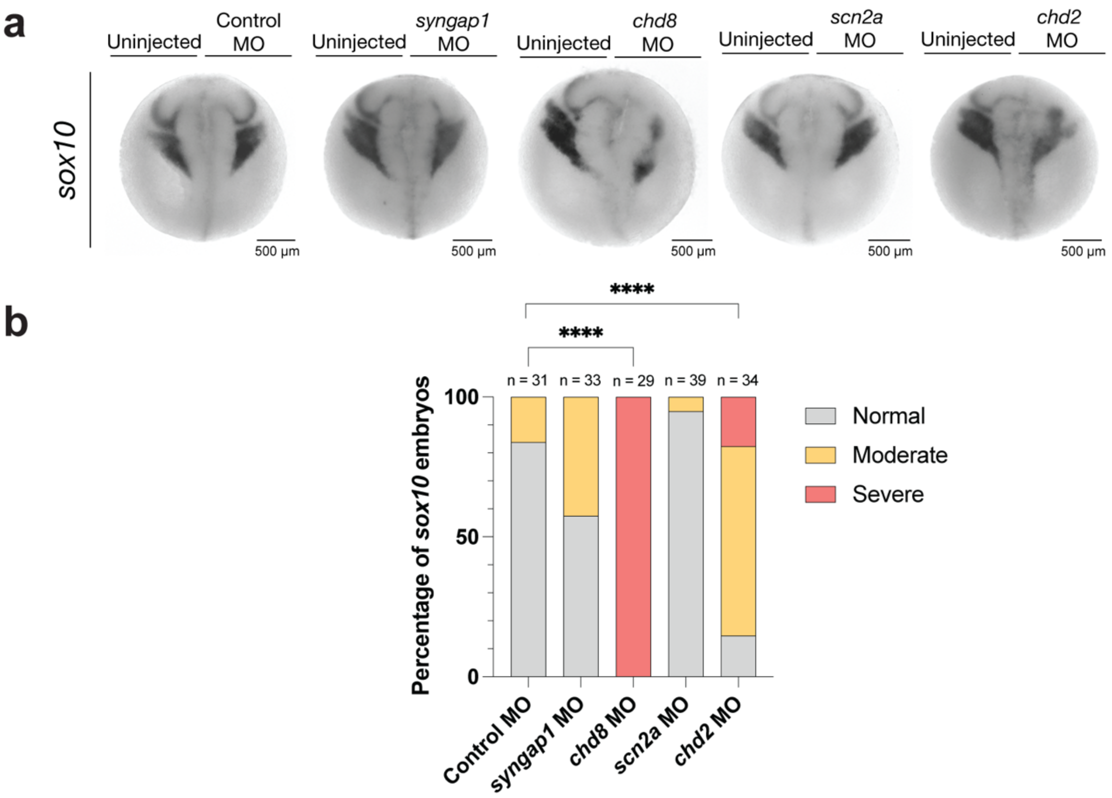

Supplementary Figure 6. hcASD genes *chd8* and *chd2* are required for neural crest development. (a) Perturbation of hcASD genes *syngap1*, *chd8*, *scn2a* or *chd2* in early (NF stage 20) embryos. (b) Proportion of severe, moderate, and normal embryo quantification by target gene. A Fisher's exact test was performed to compare the proportions to the control condition, and p-values were corrected for multiple comparisons. ****$p < 0.0001$. There is an increased proportion of moderate and severe neural crest phenotypes in *chd8* and *chd2* conditions compared to controls. There is a slight increase in the proportion of moderate phenotypes in *syngap1* embryos (p-value = 0.02 before multiple comparisons correction) and no effect on *scn2a* embryos compared to controls. Source data are provided as a Source Data file.
