# Supplementary material for: Autism gene variants disrupt enteric neuron migration and cause gastrointestinal dysmotility
Source: Nat Commun. 2025 Mar 6;16:2238. doi: 10.1038/s41467-025-57342-3 (PMC11885846; doi:10.1038/s41467-025-57342-3)
Supplement: Supplementary file 2 — Description of Additional Supplementary Files [file 41467_2025_57342_MOESM2_ESM.pdf]

**Supplementary Dataset 1.** Summary tables of patient GI symptoms from Citizen Health records for individuals with variants in *SYNGAP1*, *SCN2A*, *CHD2*, *STXBP1*, or *SLC6A1*.
