# Supplementary material for: Autism gene variants disrupt enteric neuron migration and cause gastrointestinal dysmotility
Source: Nat Commun. 2025 Mar 6;16:2238. doi: 10.1038/s41467-025-57342-3 (PMC11885846; doi:10.1038/s41467-025-57342-3)
Supplement: Supplementary file 4 — Reporting Summary [file 41467_2025_57342_MOESM4_ESM.pdf]

nature portfolio

Corresponding author(s): Helen Rankin Willsey

Last updated by author(s): Nov 7, 2024

Reporting Summary

Nature Portfolio wishes to improve the reproducibility of the work that we publish. This form provides structure for consistency and transparency in reporting. For further information on Nature Portfolio policies, see our Editorial Policies and the Editorial Policy Checklist.

Statistics

For all statistical analyses, confirm that the following items are present in the figure legend, table legend, main text, or Methods section.

n/a	Confirmed	
☐	☒	The exact sample size (*n*) for each experimental group/condition, given as a discrete number and unit of measurement
☐	☒	A statement on whether measurements were taken from distinct samples or whether the same sample was measured repeatedly
☐	☒	The statistical test(s) used AND whether they are one- or two-sided<br>*Only common tests should be described solely by name; describe more complex techniques in the Methods section.*
☒	☐	A description of all covariates tested
☐	☒	A description of any assumptions or corrections, such as tests of normality and adjustment for multiple comparisons
☐	☒	A full description of the statistical parameters including central tendency (e.g. means) or other basic estimates (e.g. regression coefficient) AND variation (e.g. standard deviation) or associated estimates of uncertainty (e.g. confidence intervals)
☐	☒	For null hypothesis testing, the test statistic (e.g. *F*, *t*, *r*) with confidence intervals, effect sizes, degrees of freedom and *P* value noted<br>*Give P values as exact values whenever suitable.*
☒	☐	For Bayesian analysis, information on the choice of priors and Markov chain Monte Carlo settings
☒	☐	For hierarchical and complex designs, identification of the appropriate level for tests and full reporting of outcomes
☒	☐	Estimates of effect sizes (e.g. Cohen's *d*, Pearson's *r*), indicating how they were calculated

Our web collection on statistics for biologists contains articles on many of the points above.

Software and code

Policy information about availability of computer code

Data collection	no software was used for data collection
Data analysis	R (v4.4.0), Seurat (v5.0.3), Prism (v10.1.1), CellRanger (v.6.0.1)

For manuscripts utilizing custom algorithms or software that are central to the research but not yet described in published literature, software must be made available to editors and reviewers. We strongly encourage code deposition in a community repository (e.g. GitHub). See the Nature Portfolio guidelines for submitting code & software for further information.

Data

Policy information about availability of data

All manuscripts must include a data availability statement. This statement should provide the following information, where applicable:

- Accession codes, unique identifiers, or web links for publicly available datasets
- A description of any restrictions on data availability
- For clinical datasets or third party data, please ensure that the statement adheres to our policy

The hcASD gene list is publically available. Publicly available human fetal gut scRNA-seq data is accessible from ArrayExpress: E-MTAB-9489. Ciitizen® medical record data is available with proper IRB approval from Ciitizen® (www.ciitizen.com). Approved researchers can obtain the Simons Searchlight population dataset described in this study by applying at https://base.sfari.org

Research involving human participants, their data, or biological material

Policy information about studies with human participants or human data. See also policy information about sex, gender (identity/presentation), and sexual orientation and race, ethnicity and racism.

Reporting on sex and gender	No analyses were conducted based on sex or gender
Reporting on race, ethnicity, or other socially relevant groupings	No analyses were conducted based on socially relevant groupings
Population characteristics	*Describe the covariate-relevant population characteristics of the human research participants (e.g. age, genotypic information, past and current diagnosis and treatment categories). If you filled out the behavioural & social sciences study design questions and have nothing to add here, write "See above."*
Recruitment	Data from Ciitizen and Simons Searchlights
Ethics oversight	UCSF IRB office

Note that full information on the approval of the study protocol must also be provided in the manuscript.

Field-specific reporting

Please select the one below that is the best fit for your research. If you are not sure, read the appropriate sections before making your selection.

☒ Life sciences ☐ Behavioural & social sciences ☐ Ecological, evolutionary & environmental sciences

For a reference copy of the document with all sections, see nature.com/documents/nr-reporting-summary-flat.pdf

Life sciences study design

All studies must disclose on these points even when the disclosure is negative.

Sample size	Sample size was determined based on similar studies in the field
Data exclusions	For Ciitizen data, individuals with ‘benign’ variants in a hcASD gene were excluded. Individuals with a ‘variant of uncertain significance’ in a hcASD gene in addition to a ‘pathogenic’ variant in a non-ASD gene were also excluded.
Replication	All experiments were performed using at least 3 biological replicates. Experiments were repeated at least two times for reproducibility and were successful
Randomization	Embryos and tadpoles were chosen at random for injections and drug treatments
Blinding	No blinding was performed in this study. Gut motility assay analyses were performed automatically with a FIJI macro.

Reporting for specific materials, systems and methods

We require information from authors about some types of materials, experimental systems and methods used in many studies. Here, indicate whether each material, system or method listed is relevant to your study. If you are not sure if a list item applies to your research, read the appropriate section before selecting a response.

Materials & experimental systems

n/a	Involved in the study	
☐	☒	Antibodies
☐	☒	Eukaryotic cell lines
☒	☐	Palaeontology and archaeology
☐	☒	Animals and other organisms
☒	☐	Clinical data
☒	☐	Dual use research of concern
☒	☐	Plants

Methods

n/a	Involved in the study	
☒	☐	ChIP-seq
☒	☐	Flow cytometry
☒	☐	MRI-based neuroimaging

Antibodies

Antibodies used	Acetylated alpha tubulin (Abcam ab179484), Dyrk1a (R and D Systems AF5407), ARL13B (ProteinTech 17711-1-AP)
Validation	https://www.abcam.com/products/primary-antibodies/alpha-tubulin-acetyl-k40-antibody-epr16772-ab179484.html<br>https://www.rndsystems.com/products/human-rat-dyrk1a-antibody_af5407#product-details<br>https://www.ptglab.com/products/ARL13B-Antibody-17711-1-AP.htm

Eukaryotic cell lines

Policy information about cell lines and Sex and Gender in Research

Cell line source(s)	hTERT RPE-1 cells (hTERT-immortalized retinal pigment epithelial cells, ATCC CRL-4000)
Authentication	Not authenticated
Mycoplasma contamination	Mycoplasma testing quarterly always negative
Commonly misidentified lines (See ICLAC register)	*Name any commonly misidentified cell lines used in the study and provide a rationale for their use.*

Animals and other research organisms

Policy information about studies involving animals; ARRIVE guidelines recommended for reporting animal research, and Sex and Gender in Research

Laboratory animals	Xenopus laevis and Xenopus tropicalis
Wild animals	no wild animals were used
Reporting on sex	animals of both sexes were used in all studies
Field-collected samples	no field-collected samples were used
Ethics oversight	UCSF IACUC-approved study

Note that full information on the approval of the study protocol must also be provided in the manuscript.

Plants

Seed stocks	*Report on the source of all seed stocks or other plant material used. If applicable, state the seed stock centre and catalogue number. If plant specimens were collected from the field, describe the collection location, date and sampling procedures.*
Novel plant genotypes	*Describe the methods by which all novel plant genotypes were produced. This includes those generated by transgenic approaches, gene editing, chemical/radiation-based mutagenesis and hybridization. For transgenic lines, describe the transformation method, the number of independent lines analyzed and the generation upon which experiments were performed. For gene-edited lines, describe the editor used, the endogenous sequence targeted for editing, the targeting guide RNA sequence (if applicable) and how the editor was applied.*
Authentication	*Describe any authentication procedures for each seed stock used or novel genotype generated. Describe any experiments used to assess the effect of a mutation and, where applicable, how potential secondary effects (e.g. second site T-DNA insertions, mosiacism, off-target gene editing) were examined.*
